# Health-Related Quality of Life in Young Adults With Perinatal HIV After Transfer to Adult Health Care in the Netherlands

**DOI:** 10.1097/QAI.0000000000003526

**Published:** 2024-11-05

**Authors:** Annouschka Weijsenfeld, Linda van der Knaap, Jane Sattoe, AnneLoes van Staa, Clementien Vermont, Jeannine F. J. B. Nellen, Dasja Pajkrt

**Affiliations:** a Department of Paediatric Infectious Diseases, Amsterdam University Medical Centers, University of Amsterdam, Amsterdam, the Netherlands;; b Department of Internal Medicine, Division of Infectious Diseases, Amsterdam University Medical Centers, University of Amsterdam, Amsterdam, the Netherlands;; c Erasmus MC- Sophia Children's Hospital, Rotterdam, the Netherlands; and; d Rotterdam University of Applied Sciences, Rotterdam, the Netherlands.

**Keywords:** HIV, young adults, health-related quality of life, adherence

## Abstract

**Introduction:**

Health-related quality of life (HRQoL) in adult people with HIV is lower than that of the general population. Previously, no differences were detected in HRQoL of Dutch children with perinatal HIV (PHIV) compared with norm groups. In this study, we compared HRQoL of PHIV young adults (PHIV-YA, aged 18–30 years) with 2 norm groups, the healthy Dutch YA population and YA with various chronic conditions.

**Methods:**

Participants received questionnaires on HRQoL, adherence, and demographics. Additional social and health care-related variables were collected from patients' medical files. We explored correlations between HRQoL and demographic characteristics. Effect sizes (ES, Hedge*s 'g* with confidence intervals) were calculated to quantify the difference between PHIV-YA and norm groups.

**Results:**

Of 81 participants, 53 filled out the questionnaires. Compared with the healthy Dutch YA population, PHIV-YA aged 18–30 years had significantly lower HRQoL scores in the school/work subscale. PHIV-YA aged 26–30 years had significantly lower total, physical and psychosocial HRQoL scores as well. Participants in the older age category had lower HRQoL scores throughout all subcategories as compared with the younger age group. For PHIV-YA aged 18–25 years, lower scores on the school/work subscale were correlated with substance use and being born outside the Netherlands.

**Conclusions:**

PHIV-YA had low HRQoL scores in school/work functioning compared with the healthy Dutch YA population. The circumstances driving these outcomes are likely to be multidimensional, including HIV infection, social background, and challenges in growing up with a chronic condition.

## Abstract 2

Supplemental Digital Content is Available in the Text.

## INTRODUCTION

A growing number of children with perinatal HIV (PHIV) survive into adulthood after introduction of effective combination antiretroviral therapies (ART).^1^ Although clinical health outcomes have improved drastically under ART, health-related quality of life (HRQoL) in the total population of adult people with HIV (PWH) is lower than that of the general population. Poor outcomes are specifically seen in measures of anxiety and depression, even in cases of a well-controlled HIV infection.^2,3^ Low mental HRQoL among Dutch PWH was also observed in comparison with people living with other chronic conditions such as diabetes mellitus type 1 or 2 or rheumatoid arthritis.^4^ Studies reporting on the influence of age, sex, educational level, and ethnic origin on HRQoL in PWH are contradictive.^2–5^

Studies evaluating the HRQoL of adolescents and young adults (YA) living with HIV reported remarkable results compared with those in adult PWH. A Dutch study on HRQoL in children and adolescents (aged 8–18 years) with PHIV showed no differences in the mean HRQoL subscales as compared with both a socioeconomically matched control group and the general Dutch population. On the emotional scale, PHIV children aged 8–12 years scored even higher compared with both control groups.^6^ Furthermore, a Spanish study in PHIV youth (aged 18–29 years) found lower physical health scores compared with both matched HIV controls and the Spanish general youth population. Mental health scores of PHIV youth were lower than those of the Spanish general youth population as well. Despite these results, overall HRQoL outcomes were relatively good and fell within the normal range.^7^ In children, adolescents, and YA with PHIV, HIV-related events that can affect physical and mental well-being are often present. In a French study, half of the participating adolescents with an age between 14 and 20 years reported HIV-related life stressors with the death of a parent being the most important stressor. Most in this study reported concerns about the disclosure of their HIV status, difficulty with adherence to treatment and follow-up, and grief regarding their HIV status.^8^ Although medium to high levels of life satisfaction were reported in 63% of the participants, 46% had psychiatric symptomatology.

Young adulthood is a period of development regarding cognition, identity, social relations, and sexuality.^9,10^ During this turbulent period, quality of life in young adults with PHIV (PHIV-YA) might be negatively influenced by having to deal with a chronic condition that (additionally) is surrounded by stigma. Although previous research found no differences in HRQoL of PHIV adolescents compared with matched controls or the general Dutch population, outcomes might be different for PHIV as they transition into adulthood. The Spanish study in PHIV aged 18–29 years found lower mental and physical HRQoL scores as compared with the general youth population.^7^ Research on the HRQoL of Dutch young adults aged >18 years with PHIV is scarce.

Therefore, this study aims to evaluate HRQoL of YAs aged between 18 and 30 years with PHIV in the Netherlands, and explore associations between health care and demographic characteristics and HRQoL outcomes.

## METHODS

We performed a multicenter cross-sectional questionnaire study. Participants were recruited from Dutch adult HIV treatment centers that treat PHIV-YA.

### Population

All patients with PHIV who transferred from pediatric to adult care in the Netherlands who had at least 1 visit in adult care were eligible to be included in the study. Participants received questionnaires on HRQoL, adherence, and demographics. An exclusion criterion was not being sufficiently proficient to read or write in the Dutch language.

### Recruitment and Consent

Specialized nurses from all 4 Dutch pediatric HIV-treatment centers identified the number of individuals who had been transferred to various adult treatment centers. Eligible individuals were informed about the study and asked to participate by their HIV specialist nurse during their visit at the outpatient clinic. In case an outpatient clinic visit was not scheduled within 3 months after the start of the study, eligible participates were approached by telephone. Those who consented to participate received an e-mail invitation with a link to an electronic informed consent form and the questionnaire. Participants gave permission to the specialized nurse to collect additional health care and demographic information from their patient file. Questionnaires became available to the participants after they signed the informed consent form. Participants received a gift certificate of €15 as a reimbursement of expenses.

### Instruments

The PedsQL 4.0 young adult (PedsQL-YA)^11^) questionnaire includes validated scales on HRQoL. The Dutch version has adequate psychometric scores and can be used for YA aged 18–30 years (Cronbach alphas 0.77–0.94).^12^ The questionnaire includes 23 items in 4 subscales: physical health (8 items), emotional functioning (5 items), social functioning (5 items), and school/work functioning (5 items). Items are rated on a 5-point Likert scale (0 = never a problem, 1= almost never a problem, 2 = sometimes a problem, 3 = often a problem, 4 = nearly always a problem). Each answer is reversed scored and rescaled to a 0–100 scale (0 = 100, 1 = 75, 2 = 50, 3 = 25, and 4 = 0). Higher scores indicate a higher HRQoL. A psychosocial health score is computed as the sum of the scores of emotional, social, and school/work scales. The scores from different subscales are divided by the number of items in the subscale. We compared PedsQL-YA scores and calculated effect sizes for PHIV-YA to 2 norm groups (healthy and various chronic conditions, eg, asthma, psychiatric disorders, gastroenterology, and skin disease) for age groups 18–25 and 26–30 years.^12^

We assessed self-reported adherence by 4 questions about adherence rated on a 5–6-point Likert scale. This questionnaire can predict an undetectable HIV viral load (HIV-VL) comparable with assessment of adherence through pharmacy refill [positive predictive value (PPV), 95%; negative predictive value (NPV), 13%].^13^

The following health care and demographic variables were collected through a separate questionnaire: country of birth, country of birth of both parents, current educational level, employment, income, family situation, ART use yes/no, substance use, and age at transfer to an adult treatment center.

Additional information on demographic and health care-related variables was collected systematically from patient files by the HIV specialist nurse and included year of birth, sex, history of contact with social services yes/no, death of 1 or both biologic parents, time of transfer to adult care, family situation at transfer, period of lost to follow-up >12 months yes/no, ART use yes/no, last viral load (VL) in pediatric care/last VL in adult care, CDC classification at first visit pediatric care, and (nadir) CD4^+^ T-cell count. Viral suppression was defined by an HIV-RNA measurement <50 copies/mL.

The questionnaires were available to health care providers and to participants through CASTOR.^14^

The study was assessed by the committee for medical ethics of the Amsterdam UMC and local medical ethics committees of participating centers and was no subject to the Medical Research Involving Human Subjects Act.

### Statistical Analyses

Sample characteristics were described by median (IQR) and number and percentages where appropriate. To compare characteristics between responders and nonresponders, we used the Mann–Whitney *U* test for non-normally distributed continuous variables and χ^2^ or the Fisher exact tests for proportions. Effect sizes [*Hedges G/*confidence interval (CI) for groups with different sample sizes] were calculated to describe the magnitude of differences in sum scores [Mean (SD)] on the total, physical, emotional, social, school, and psychosocial domains of the PedsQL-YA between PHIV-YA, YA with various (self-reported) chronic health conditions, and Dutch YA norm data. An effect size of >0.5 (medium to large) was considered significant and clinically relevant.^15–17^

A score of >1 SD less than the sample mean of the norm population represented an impaired HRQoL.^18^ For the calculation of adherence, a participant was classified as “adherent” if they had a composite Likert score of 20–22 points.

Our data did not meet the assumptions of a linear regression. Therefore, we explored correlations between demographic variables and total HRQoL scores and between demographic variables and school/work HRQoL scores. Variables potentially correlated with HRQoL outcomes were sex, age, education level, parental loss, country of birth, and substance use. Correlations between HRQoL scores with a normal distribution and variance and binary variables were explored using Point Biserial tests (*r*). Correlations between HRQoL scores and continuous variables with a normal distribution and variance were explored using Pearson *r* (*r*). Correlations between HRQoL scores with a non-normal distribution and/or variance and binary variables were explored using Mann–Whitney *U* tests. The correlation coefficient for the Mann–Whitney *U* test was calculated using the following equation: *r* = Z: Sqrt N.^19^ Spearman Rho tests (*ρ*) were used to assess correlations between HRQoL scores (with a non-normal distribution and/or variance) and continuous or ordinal variables. A correlation coefficient with a *P* < 0.05 and a correlation coefficient > ±0.4 (moderate to strong) was considered significant and clinically relevant.^20^

Correlations between having an impaired total HRQoL and an impaired school/work HRQoL were explored using χ^2^ or Fisher exact for dichotomous variables (correlation coefficient Phi).

Statistics were performed using IBM SPSS Statistics for Windows, Version 28.0. Armonk, NY: IBM Corp, Social Science Statistics^21^ and Effect Size Calculators.^22^

## RESULTS

**FIGURE 1.**
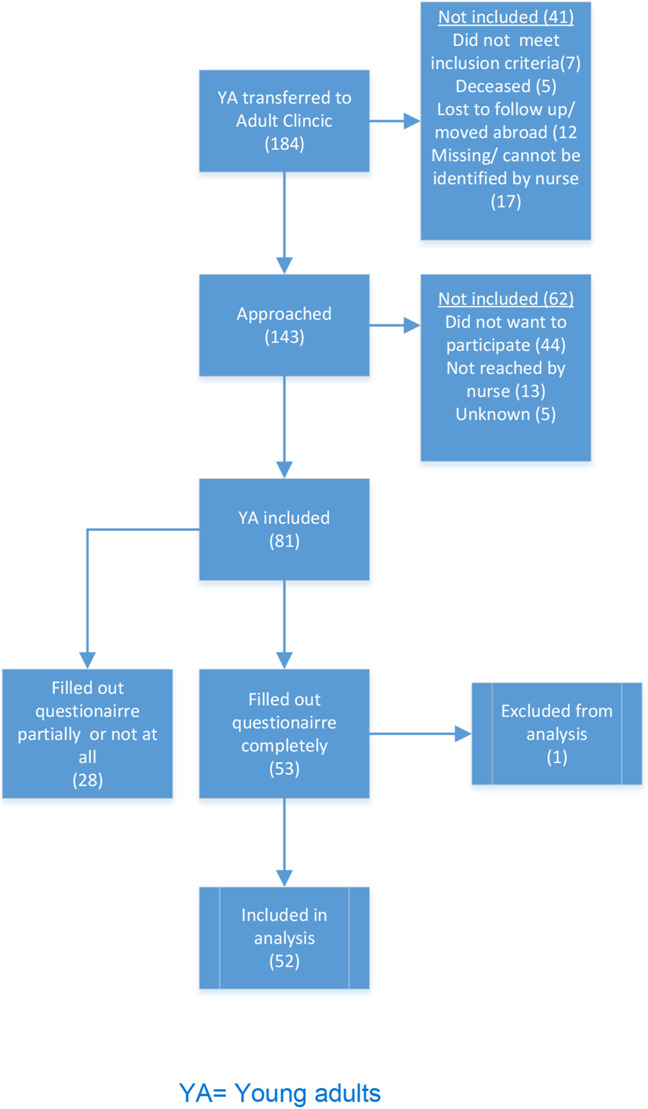
Flowchart inclusion.

Between November 2020 and April 2022, 143 eligible participants were contacted to participate in this study. Of those contacted, 81 (57%) consented to participate in the study, and of these, 53 (65%) filled out the questionnaires on HRQoL, adherence, and demographics themselves. One extreme outlier on HRQoL scores was removed. The final number of participants who were included in the analyses was 52 (64%). The most commonly reported reasons for nonparticipation were “too busy,” “do not want to talk about HIV,” and “privacy reasons.” The total response rate was 36% (Fig. 1).

### Demographics

Responders (N = 52) were more likely to have an undetectable HIV-VL compared with nonresponders (N = 28) (*P* = 0.02). They did not differ significantly in other available clinical and demographic characteristics (see Table 1, Supplemental Digital Content, http://links.lww.com/QAI/C364). Of the responders, 28 (53.8%) were male, their median age at the time of the study was 23.0 years and 24 (46.2%) were born in the Netherlands. Four (7.7%) participants had a biologic mother and 11 (21.2%) a biologic father born in the Netherlands. In pediatric care, youth social services (YSS) were involved in 20 participants (38.5%), 7 (13.5%) were adopted, and of 12 participants (23.1%), 1 or both parents were deceased during childhood (Tables 1 and 2).

**TABLE 1.** Demographic and Clinical Data of Research Population Provided by Health Care Professionals

Demographic and Clinical Characteristics	N *=* 52
Sex	
Male	28 (53.8)
Female	24 (46.2)
Age at study date	23.0 (20.0–29.0)
Time transfer, yrs	
<2	12 (23.1)
2–4	7 (13.5)
4–6	10 (19.2)
>6	23 (44.2)
HIV VL undetectable at transfer	
Yes	48 (92.3)
No	4 (7.7)
HIV VL undetectable at last visit	
Yes	50 (96.2)
No	2 (3.8)
Nadir CD4 count pretransfer	355 (220–519)
Nadir CD4 count posttransfer	530 (324–780)
CDC category at the start of care	
N (no symptoms)	11 (21.2)
A (no symptoms)	16 (30.8)
B (symptoms)	12 (23.1)
C (aids)	12 (23.1)
Missing	1 (1.9)
CD4 count at transfer	621 (444–915)
CD4 count at last visit	710 (513–865)
ART use at transfer	
Yes	48 (92.3)
No	4 (7.7)
ART use at study date (health care provider)	
Yes	50 (96.2)
No	2 (3.8)
Loss to follow-up >12 months (ever)	
Yes	6 (11.5)
No	46 (88.5)
One or both parents deceased pretransfer	
Yes	12 (23.1)
No	40 (76.9)
Adoption	
Yes	7 (13.5)
No	45 (86.5)
Any form of youth social services pretransfer	
Yes	20 (38.5)
No	32 (61.5)
Involvement of social services posttransfer	
Yes	12 (23.1)
No	35 (67.3)
Unknown	5 (9.6)
Living situation at transfer	
Biologic parents	16 (30.7)
Biologic mother	16 (30.7)
Biologic father	6 (11.5)
Foster/adoption	12 (23.1)
Other	2 (3.8)

Characteristics described by median (IQR), and number and percentages where appropriate.

**TABLE 2.** Self-Reported Demographic Data of Responders

Demographic and Clinical Characteristics	N = 52
Country of birth	
The Netherlands	24 (46.2)
Other	28 (53.8)
Country of birth mother	
The Netherlands	4 (7.7)
Other	45 (86.5)
Missing	3 (5.8)
Country of birth father	
The Netherlands	11 (21.2)
Other	38 (73.0)
Missing	3 (5.8)
Age at transfer	18.2 (18.0–18.0)
Current living situation	
Independent living (1 = ass. Living)	23 (44.2)
With parents/family	29 (55.8)
Partner	
Yes	11 (21.2)
No	34 (65.4)
Missing	7 (13.5)
Disclosure of HIV status to partner	
Yes	10 (91.0)
No	1 (9.0)
Children	
Yes	7 (13.5)
No	45 (86.5)
Educational level	
Low	11 (21.2)
Middle	31 (59.6)
High	10 (19.2)
Work	
Paid job	36 (69.2)
Volunteer work	1 (1.9)
Not working	15 (28.8)
Monthly income (after tax)	
<€1000	15 (28.8)
€1000–€2500	19 (36.5)
€2500–€5000	5 (9.6)
>€5000	1 (1.9)
Prefer not to report	12 (23.1)
ART use at study date	
Yes	2 (100)
No	—
Alcohol use ever	
Yes	29 (55.8)
No	23 (44.2)
Alcohol past week	
Yes	15 (28.8)
No	37 (71.2)
Smoking	
Yes	6 (11.5)
No	46 (88.5)
Substance use*	
Yes	11 (21.2)
No	41 (78.8)

Characteristics described by median (IQR), and number and percentages where appropriate.

* Nitrous oxide n = 2; cannabis n = 10; XTC/MDMA n = 3; cocaine n = 1; LSD n = 1; ketamine n = 1.

### Adherence

At the time of the study date, 50 participants (96.2%) had an undetectable HIV-VL, of whom 20 were adherent according to our definition. Of 32 participants who were not adherent, 2 had a detectable HIV-VL (PPV: 6.3%, NPV 100% (see Table 2, Supplemental Digital Content, http://links.lww.com/QAI/C365)).

### Health-Related Quality of Life

Norm scores differed for age groups 18–25 and 26–30 years, therefore, effect sizes were calculated separately for these age groups. PHIV-YA aged 18–25 years had overall higher HRQoL scores than Dutch YA with chronic health conditions. However, we only observed significant differences on the subscale of social functioning (*g* = 0.63, CI: 0.22 to 1.06). For PHIV-YA aged 26–30 years, no significant differences were observed on any subscale compared with those of Dutch YA with chronic health conditions (Table 3).

**TABLE 3.** Health-Related Quality of Life Scores and Effect Sizes for Young Adults With Perinatal HIV Compared With 2 Reference Groups

Age		HIV, N = 32		Healthy, N = 310						Chronic Health Condition, N = 75					
		M (SD)		M (SD)		*g*		CI		M (SD)		*g*		CI	
18–25															
Total score		81.3 (10.5)		85.9 (11.2)		0.41		0.05 to 0.78		75.2 (15.1)		0.43		0.02 to 0.86	
Physical health		86.4 (15.8)		90.2 (12.5)		0.30		−0.07 to 0.66		77.8 (20.5)		0.44		0.03 to 0.86	
Psychosocial health		79.6 (11.1)		83.6 (12.7)		0.32		−0.68 to 0.05		73.8 (14.8)		0.42		0.00 to 0.84	
Emotional functioning		76.3 (16.1)		78.4 (17.7)		0.12		−0.48 to 0.24		69.7 (18.1)		0.37		−0.04 to 0.79	
Social functioning		89.4 (11.8)		88.4 (13.7)		0.07		−0.29 to 0.44		79.2 (17.4)		**0.63**		**0.22 to 1.06**	
School/work functioning		73.3 (14.1)		84.0 (14.3)		**0.75** *		**0.38 to 1.12**		72.7 (16.9)		0.04		−0.38 to 0.45	
26–30		N = 20		N = 202						N = 62					
Total score		76.8 (14.1)		85.9 (11.9)		**0.75**		**0.29 to 1.22**		78.4 (16.9)		0.1		−0.60 to 0.40	
Physical health		80.6 (18.4)		88.7 (13.8)		**0.57**		**0.11 to 1.03**		78.0 (26.6)		0.1		−0.40 to 0.61	
Psychosocial health		75.5 (14.8)		84.3 (13.0)		**0.67**		**0.20 to 1.13**		78.6 (15.8)		0.2		−0.70 to 0.31	
Emotional functioning		70.8 (19.9)		78.7 (17.5)		0.44		−0.91 to 0.02		75.4 (19.5)		0.23		−0.74 to 0.27	
Social functioning		87.5 (15.2)		89.3 (12.8)		0.14		−0.60 to 0.32		83.6 (16.6)		0.24		−0.27 to 0.74	
School/work functioning		68.3 (18.3)		85.0 (14.6)		**1.12**		**0.64 to 1.59**		76.7 (19.1)		0.44		−0.95 to 0.07	

Effect sizes were calculated through Hedges *g*.

* Bold numbers represent a clinically significant result.

Comparison with the healthy Dutch norm population showed a significant lower HRQoL score in the school/work subscale for both PHIV aged 18–25 years and PHIV aged 26–30 years (*g* = 0.75, CI: 0.38 to 1.12/*g* = 1.12, CI: 0.64 to 1.59, respectively). In those aged 26–30 years, scores on total, physical, and psychosocial subscales were significantly lower than the healthy Dutch norm population as well (*g* = 0.75, CI: 0.29 to 1.22*, g* = 0.57, CI: 0.11 to 1.03 and *g* = 0.67, CI: 0.20 to 1.13, respectively) (Table 3).

Participants in the older age category had lower HRQoL scores throughout all subcategories than the younger age group.

The proportion of participants with and impaired total HRQoL was 25% (n = 8) for those aged 18–25 years and 35% (n = 7) for those aged 26–30 years. An impaired score on the school/work subscale was seen in 40% (n = 13) and 55% (n = 11), respectively (Table 4).

**TABLE 4.** Number and % of Young Adults With Perinatal HIV With an Impaired* Health-Related Quality of Life

	Impaired HRQoL
Age group 18–25	N = 32
Total score	8 (25)
Physical health	6 (19)
Psychosocial health	7 (22)
Emotional functioning	8 (25)
Social functioning	6 (19)
School/work functioning	13 (40)
All domains	2 (6)
Age group 26–30	N = 20
Total score	7 (35)
Physical health	6 (30)
Psychosocial health	8 (40)
Emotional functioning	7 (35)
Social functioning	5 (25)
School/work functioning	11 (55)
All domains	4 (20)

* An impaired HRQoL was defined as a score of >1 SD less than the mean of the reference population of healthy Dutch young adults.

### Correlations With Health-Related Quality of Life

In the total study group, none of the included independent variables showed a significant correlation with the outcome variables.

For PHIV-YA aged 18–25 years, lower scores on school/work HRQoL were significantly correlated with being born outside the Netherlands (*r* = −0.44, *P* = 0.013, z =−2.49) and substance use (*r =* −0.41, *P* = 0.020, z = −2.32). Furthermore, being born outside the Netherlands and substance use were correlated with impaired school/work HRQoL (*phi* = 0.47, *P* = 0.012 and *phi* = 0.40, *P* = 0.038, respectively).

For PHIV-YA aged 26–30 years, none of the included independent variables showed a significant correlation with the outcome variables.

## DISCUSSION

This study assessed HRQoL in PHIV-YA aged 18–25 and 26–30 years. Although both age categories showed significant differences in work/school-related HRQoL compared with the healthy YA population, the differences were larger in those aged 26–30 years. Furthermore, both those aged 18–25 years and those aged 26–30 years had high proportions of impaired work/school functioning. These outcomes are in contrast with previous research in the Netherlands where PHIV children, adolescents, and YA did not perform worse on any of the HRQoL subscales compared with matched controls or the general population.^6,23^ Still, in 1 study, the proportion of children with an impaired school functioning was significantly higher than that of the general population.^6^ The authors suggested poorer performance on several cognitive functions that was found in an earlier study as an explanation for this increased proportion of children with impaired school functioning.^24^ This suggestion is strengthened by a study in PHIV young people aged 12–21 years that found scores less than the norm population on cognitive performance in verbal learning and recall, more present in those with a past CDC-C event.^25^ Furthermore, cognitive impairment in working memory, executive functioning, and processing speed in PHIV children and adolescents was described.^26^ Besides this, cognitive fatigue, relating to memory and keeping attention, could play a role in the low school/work scores for a Dutch study in 14 children and adolescents found relatively low (not significant) scores in the cognitive fatigue scale compared with HIV-negative matched controls and the general population.^27^ It is plausible that these factors are also present in our study population, as part of our study population is also represented in these studies. Therefore, our outcomes can indeed be because of school functioning problems related to cognitive impairment and fatigue that extend to experiencing professional work-related problems. In addition, lowered physical health in those aged 26–30 years may also affect school/work performances. Research highlighted the importance of given support by HIV specialist nurses and occupational health physicians in case of work-related problems. An occupational health physician can offer the employer guidance on employability while maintaining confidentiality regarding HIV status.^28^

Lower school/work HRQoL was correlated with being born outside the Netherlands in those aged 18–25 years. In the Dutch PHIV population, ART was significantly delayed in children originating from Sub-Saharan Africa compared with children born in the Netherlands.^29^ They are possibly disadvantaged because several studies showed that deferred ART in children is associated with poorer neurocognitive performance, which remained after starting ART.^30^ However, we could not identify this association in the older age group, who were born before 1998. They are more likely to have a delayed start of ART, together with exposure to less potent and more complex ART regimes independent of birth country. In our population, ART start and history could influence school/work HRQoL.

Lower scores on the school/work subscale were correlated with substance use (mainly cannabis, Table 1) in those aged 18–25 years, but not in those aged 26–30 years. Cannabis use is associated with poorer academic performance and deficits in learning, working and memory function, and dependent on the frequency of use.^31,32^ In our population, 5 of the 7 participants in the 18–25 years age group reported substance use, which occurred more than 1 month ago, and we did not assess the frequency of substance use. Therefore, we must be cautious when interpreting this correlation.

Both PHIV age groups showed no significantly worse outcomes in social and emotional subscales compared with the reference groups, and even report better outcomes in social functioning compared with other chronic conditions. Probably, the impact on social participation of conditions such as asthma or psychiatric disorders is higher than the impact of HIV. The earlier mentioned English study^25^ found no differences in anxiety and depression in PHIV young people and controls. The absence of differences is notable because multiple studies in the adult population of PWH showed worse mental HRQoL compared with other chronic conditions^4^ and the general population.^2,3^ The 2 latter studies specifically reported on high occurrence of anxiety and depression. Possibly, with older age, HIV-related stigma and disclosure concerns have a larger impact on emotional well-being of PHIV YA. Indeed for those aged 26–30 years, emotional subscale scores are lower than for those aged 18–24 years, and contribute to a low psychosocial health composite score with a high magnitude compared with the healthy population. Low mental health scores compared with the general population were also reported before in PHIV YA aged 18–30 years.^7^ In addition, increasing age predicted mental health decrease driven by perceived stigma in PHIV YA.^33^

In assessing HRQoL outcomes, we have to consider other variables that can effect these outcomes, beside the consequences of solely PHIV. First of all, several studies that assessed school functioning and cognitive functions in PHIV and in HIV-exposed uninfected (HEU) children or matched controls also found worse outcomes in those who were HEU compared with national norms.^24,34^ Thus aside from the HIV infection, socioeconomic background is most likely of influence on school/work functioning.

Second, in our study, the reference group of other chronic conditions had outcomes similar to PHIV-YA on school work functioning in those aged 18–25 years. Multiple educational consequences of growing up with a chronic condition are recognized, such as lower academic performance and attainment and worse outcomes related to absenteeism compared with healthy students.^35^ In our study population, we did not find a relationship between school/work HRQoL and educational level.

Levels of viral suppression were high despite low levels of adherence according our definition. The adherence questionnaire does not provide useful information in assessing whether an efficient level of adherence is accomplished to achieve undetectable HIV-VL.

In summary, PHIV-YA have low HRQoL scores in school/work functioning, especially in the 26–30 years age group. More emotional problems were reported in this older age group. The circumstances driving these outcomes are likely to be multidimensional, including HIV infection, social background, and challenges in growing up with a chronic condition.

### Practical Recommendations

- School performance should be a recurrent topic during regular consultations at the pediatric HIV department. Health care professionals (HCP) can help parents in finding their way to support systems in case additional support is needed.^34^
- HCP at the pediatric department should inform parents about the influence of HIV on possible learning challenges. In that way, PHIV children can receive additional support as early as possible, to fully develop their potential.
- Ideally teachers and school counselors are informed about the HIV diagnoses and possible consequences for school and academic performance.
- In adult care, HCP should evaluate school/work participation during their regular consultation. In case of school/work-related problems, people can be referred to their school counselor or occupational health physician.
- For practical recommendations to improve work-related care for HIV, HCP can consult national multidisciplinary guidelines such as those existing in the Netherlands “HIV and Employment.”^36^

### Limitations

Our study has some limitations. Firstly, the PedsQL-YA is validated for persons aged 18–30 years. In our cohort, there were 5 participants aged >30 years (31–37). We decided not to exclude them, for we believe that they are representable for the composition of the PHIV population in general. Sensitivity analyses excluding these participants showed a decrease [from 70.8 (19.9) to 68.9 (20.3)] for emotional HRQoL resulting in a significant effect size compared with the healthy Dutch norm population (E.S. from 0.44 to 0.55). This supports our hypothesis that with older age, HIV-related stigma and disclosure concerns potentially have a larger impact on emotional well-being of PHIV YA.

Secondly, although HRQoL subscores for physical and social health did not follow a normal distribution, we used the mean (SD) to compare our results with the result of the norm groups. This non-normal distribution possibly biased the results for these nonsignificant subscores.

Furthermore, our study population is not completely representative for the Dutch PHIV-YA population. Undetectable HIV-VL levels were seen in 96.2%, whereas a recent article including all registered PHIV-YA in the Netherlands reported undetectable HIV-VL levels of only 82.6%.^37^ Although viral suppression is not a marker for emotional well-being, depressive symptoms are associated with nonadherence in PWH,^38^ and, therefore, HRQoL in our cohort might be overestimated.

Also, our study was conducted between November 2020 and September 2022, and, therefore, partly took place during the COVID-19 pandemic that came with stringent measures that impacted daily life, varying from social distancing to total lockdowns. These measures had a negative effect on emotional well-being of young people in the Netherlands and the rest of the world. However, feelings of solidarity and reduced stress were also reported.^39^

Finally, we did not include questions on perceived HIV-related stigma and, therefore, cannot evaluate the impact of experienced stigma on emotional and social well-being. Although no differences were found on these subscales compared with the healthy Dutch youth population, older PHIV-YA did score lower on emotional HRQoL as compared with younger PHIV-YA. Indeed studies describe the worries and stress caused by having to deal with disclosure concerns and stigma experienced by PHIV YA when engaging in romantic relationships.^40,41^

Whether and how stigma affects the lives of PHIV-YA is of interest and worth to explore through future research, because social and internalized HIV stigma is still high in the Netherlands and among all age groups of PWH.^42–44^

## Supplementary Material

SUPPLEMENTARY MATERIAL
