# Supplementary material for: Health-Related Quality of Life in Young Adults With Perinatal HIV After Transfer to Adult Health Care in the Netherlands
Source: J Acquir Immune Defic Syndr. 2024 Nov 5;97(5):514–21. doi: 10.1097/QAI.0000000000003526 (PMC11540273; doi:10.1097/QAI.0000000000003526)
Supplement: SUPPLEMENTARY MATERIAL [file qai-97-514-s001.docx]

| Supplemental table 1: Demographic and clinical data of responders and non-responders. | | | | |
| --- | --- | --- | --- | --- |
| *Demographic and clinical characteristics* |  | *Responders*  *N=52* | *Non-responders*  *N= 28* | *P-value* |
| Sex | *Male*  *Female* | 28 (53.8)  24 (46.2) | 14 (50)  14 (50) | .88 |
| Age at study date |  | 23.0 (20.0-29.0) | 23.0 (21.0-26.8) | .71 |
| Time transfer | *< 2 years*  *2-4 years*  *4-6 years*  *> 6 years* | 12 (23.1)  7 (13.5)  10 (19.2)  23 (44.2) | 3 (10.7)  8 (28.6)  5 (17.9)  12 (42.9) | .31 |
| HIV VL undetectable at transfer | *Yes*  *No* | 48 (92.3)  4 (7.7) | 23 (82.1)  5 (17.9) | .27 |
| HIV VL undetectable at last visit | *Yes*  *No* | 50 (96.2)  2 (3.8) | 22 (78.6)  6 (21.4) | **.02*** |
| Nadir CD4 count pre-transfer |  | 355 (220-519) | 394 (217-570) | .62 |
| Nadir CD4 count post-transfer |  | 530 (324-780) | 602 (390-700) | .95 |
| CDC category at the start of care | *N (no symptoms)*  *A (no symptoms)*  *B (symptoms)*  *C (aids)*  *Missing* | 11 (21.2)  16 (30.8)  12 (23.1)  12 (23.1)  1 (1.9) | 3 (10.7)  10 (35.7)  11 (39.3)  4 (14.3)  - | .41 |
| CD4 count at transfer |  | 621 (444-915) | 700 (480-847) | .96 |
| CD4 count at last visit |  | 710 (513-865) | 680 (501-780) | .52 |
| ART use at transfer | *Yes*  *No* | 48 (92.3)  4 (7.7) | 27 (96.4)  1 (3.6) | .65 |
| ART use at study date (health care provider) | *Yes*  *No* | 50 (96.2)  2 (3.8) | 26 (92.9)  2 (7.1) | .61 |
| Loss to follow up > 12 months (ever) | *Yes*  *No* | 6 (11.5)  46 (88.5) | 3 (10.7)  25 (89.3) | 1.00 |
| One or both parents deceased pre- transfer | *Yes*  *No* | 12 (23.1)  40 (76.9) | 9 (32.1)  19 (67.9) | .43 |
| Adoption | *Yes*  *No* | 7 (13.5)  45 (86.5) | 0  28 (100) | .066 |
| Involvement of social services pre-transfer | *Yes*  *No* | 20 (38.5)  32 (61.5) | 10 (35.7)  18 (64.3) | 1.00 |
| Involvement of social services post-transfer | *Yes*  *No*  *Unknown* | 12 (23.1)  35 (67.3)  5 (9.6) | 8 (28.6)  18 (64.3)  2 (7.1) | .87 |
| Living situation at transfer | *Biological parents*  *Biological mother*  *Biological father*  *Foster/adoption*  *Other* | 16 (30.7)  16 (30.7)  6 (11.5)  12 (23.1)  2 (3.8) | 6 (21.4)  14 (50)  2 (7.1)  3 (10.7)  3 (10.7) | .25 |

We used the Mann-Whitney U test for medians and Chi- Square or the Fisher exact test for proportions. ^*^ Bold number represents a significant result.
