# Supplementary material for: Health-Related Quality of Life in Young Adults With Perinatal HIV After Transfer to Adult Health Care in the Netherlands
Source: J Acquir Immune Defic Syndr. 2024 Nov 5;97(5):514–21. doi: 10.1097/QAI.0000000000003526 (PMC11540273; doi:10.1097/QAI.0000000000003526)
Supplement: SUPPLEMENTARY MATERIAL [file qai-97-514-s002.docx]

| Supplemental table 2: self-reported adherence. | | |
| --- | --- | --- |
|  |  | N=52 |
| Q1: ‘Thinking about the past four weeks, how would you rate your ability to take all your medications as your doctor prescribed them?’ | *Very bad*  *Bad*  *Reasonable*  *Good*  *Very good*  *Excellent*  *Not sure* | 0  2  1  15  13  20  1 |
| Q2: ‘Thinking about the past four weeks, how often did you take all your HIV antiretroviral medications as your doctor prescribed them?’ | *Never*  *Sometimes*  *Quite often*  *Usually*  *All of the time* | 0  2  0  8  42 |
| Q3: ‘How many days in the past week did you take all anti-HIV medicines that were prescribed?’ | *0 days*  *1-3 days*  *3-4 days*  *5-6 days*  *All 7 days*  *Prefer not to say* | 0  0  1  4  46  1 |
| Q4: ‘When was the last time you missed any of your anti- HIV medications?’ | *Last week*  *1-2 weeks ago*  *2-4 weeks ago*  *1-3 months ago*  *>3 months ago*  *Never*  *Not sure* | 4  8  3  6  8  6  17 |
| Adherent* | *Yes*  *No* | 20  32 |

*Participants were classified as ‘adherent’ if they had the following answers: - Q1: ‘very good’ or ‘excellent’ - Q2: ‘all of the time’- Q3: ‘all 7 days’ -Q4: ‘more than three months ago’, ‘never missed’ or ‘I’m not sure.
